# Dental ceramics: An update

**DOI:** 10.4103/0972-0707.73379

**Published:** 2010

**Authors:** Arvind Shenoy, Nina Shenoy

**Affiliations:** Department of Conservative Dentistry, Bapuji Dental College and Hospital, Davangere, Karnataka, India; 1 Department of Periodontology, ABSMIDS, Mangalore - 575 003, Karnataka, India

**Keywords:** Ceramics, CADCAM, zirconia

## Abstract

In the last few decades, there have been tremendous advances in the mechanical properties and methods of fabrication of ceramic materials. While porcelain-based materials are still a major component of the market, there have been moves to replace metal ceramics systems with all ceramic systems. Advances in bonding techniques have increased the range and scope for use of ceramics in dentistry. In this brief review, we will discuss advances in ceramic materials and fabrication techniques. Examples of the microstructure property relationships for these ceramic materials will also be addressed.

## INTRODUCTION

Dental ceramics are materials that are part of systems designed with the purpose of producing dental prostheses that in turn are used to replace missing or damaged dental structures. The literature on this topic defines ceramics as inorganic, non-metallic materials made by man by the heating of raw minerals at high temperatures.[1]

Ceramics and glasses are brittle, which means that they display a high compressive strength but low tensile strength and may be fractured under very low strain (0.1%, 0.2%).

As restorative materials, dental ceramics have disadvantages mostly due to their inability to withstand functional forces that are present in the oral cavity. Hence, initially, they found limited application in the premolar and molar areas, although further development in these materials has enabled their use as a posterior long-span fixed partial prosthetic restorations and structures over dental implants.[2] All dental ceramics display low fracture toughness when compared with other dental materials, such as metals.[3]

Metal ceramic systems combine both the exceptional esthetic properties of ceramics and the extraordinary mechanical properties of metals.[4] Some metals used as restorative materials in dentistry may constitute a problem for some patients. These problems may reveal themselves as allergies,[5] gum staining[6,7] and release of metallic ions into the gingival tissue[8] and the gingival fluid.[9] These drawbacks, as well as the search for more esthetic materials by patients and dentists, have stimulated research and development of metal-free ceramic systems.

The main objective of this work is to review ceramic dental materials, including their most relevant physical and mechanical properties.

## CLASSIFICATION

Ceramics can be classified by their microstructure (i.e., amount and type of crystalline phase and glass composition).[10]

They can also be classified by the processing technique (power-liquid, pressed or machined).

## MICROSTRUCTURAL CLASSIFICATION

At the microstructural level, we can define ceramics by the nature of their composition of glass-to-crystalline ratio. There can be infinite variability of the microstructures of materials, but they can be broken down into four basic compositional categories, with a few subgroups:

- composition category 1 – glass-based systems (mainly silica),
- composition category 2 – glass-based systems (mainly silica) with fillers, usually crystalline (typically leucite or, more recently, lithium disilicate),
- composition category 3 – crystalline- based systems with glass fillers (mainly alumina) and
- composition category 4 – polycrystalline solids (alumina and zirconia).

## GLASS-BASED SYSTEMS

Glass-based systems are made from materials that contain mainly silicon dioxide (also known as silica or quartz), which contains various amounts of alumina.

Aluminosilicates found in nature, which contain various amounts of potassium and sodium, are known as feldspars. Feldspars are modified in various ways to create the glass used in dentistry. Synthetic forms of aluminasilicate glasses are also manufactured for dental ceramics.

### Composition category 2 – Glass-based systems with fillers

This category of materials has a very large range of glass–crystalline ratios and crystal types, so much so that this category can be subdivided into three groups. The glass composition is basically the same as the pure glass category.

1. The difference is that varying amounts of different types of crystals have either been added or grown in the glassy matrix. The primary crystal types today are leucite, lithium disilicate or fluoroapatite.

#### Subcategory 2.1

Low-to-moderate leucite-containing feldspathic glass – these materials have been called “feldspathic porcelains” by default. Even though other categories have a feldspathic-like glass, this category is what most people mean when they say “feldspathic porcelain.”

#### Subcategory 2.2:

High-leucite-containing (approximately 50%) glass. Again, the glassy phase is based on an aluminosilicate glass. These materials have been developed in both powder/liquid, machinable and pressable forms.

#### Subcategory 2.3:

Lithium-disilicate glass ceramic is a new type of glass ceramic introduced by Ivoclar as IPS Empress^®^ II (now called IPS e.max^®^), where the aluminosilicate glass has lithium oxide added.

### Composition category 3 – Crystalline-based systems with glass fillers

Glass-infiltrated, partially sintered alumina was introduced in 1988 and marketed under the name In-Ceram. The system was developed as an alternative to conventional metal ceramics and has met with great clinical success.

### Composition category 4 – Polycrystalline solids

Solid-sintered, monophase ceramics are materials that are formed by directly sintering crystals together without any intervening matrix to from a dense, air-free, glass-free, polycrystalline structure. There are several different processing techniques that allow the fabrication of either solid-sintered aluminous-oxide or zirconia-oxide frameworks.

### Classification based on processing technique

A more user-friendly and simplistic way to classify the ceramics used in dentistry is by how they are processed. It is important to note that all materials can be processed by varied techniques. But, in general, for dentistry, they can be classified as:

1. Powder/liquid, glass-based systems,
2. machinable or pressable blocks of glass-based systems and
3. CAD/CAM or slurry, die-processed, mostly crystalline (alumina or zirconia) systems.

#### Powder/liquid, with or without crystalline fillers

These are the porcelains that are made for veneering cores made from either metal, alumina or zirconia, but can be used for porcelain veneers on either a refractory die or platinum foil technique.

#### Manufactured blocks, with or without crystalline fillers

Vitabloc Mark II for the CEREC and pressable and machinable versions of IPS Empress are the primary materials available in this classification. These materials are ideally suited for inlay and onlay restorations, anterior crowns and veneers, and possibly bicuspid crowns. They have to be bonded and can be used full contour as there are polychromatic machinable versions.

#### CAD/CAM or slurry/die-generated mostly or all-crystalline alumina- or zirconia-based systems

Alumina materials in this classification are Procera, which is solid sintered alumina, and In-Ceram, which is glass infiltrated. These materials work well for cores for single crowns that are veneered with a powder/liquid glass-based material (porcelain).

## GLASS CERAMICS

Glass ceramics were first developed by Corning Glass Works in the late 1950s. According to McLean,[11] the first works on glass ceramics were performed by Mac Culloch, but his work did not receive much attention. Further investigations by Grossman and Adair[12,13] concluded with the development of a tetra silicic fluormica-containing ceramic system.

In principle, an article is formed while liquid and a metastable glass results on cooling. During a subsequent heat treatment, controlled crystallization occurs, with the nucleation and growth of internal crystals. This conversion process from a glass to a partially crystalline glass is called *ceraming*. Thus, a glass ceramic is a multiphase solid containing a residual glass phase with a finely dispersed crystalline phase. The controlled crystallisation of the glass results in the formation of tiny crystals that are evenly distributed throughout the glass. The number of crystals, their growth rate and thus their size are regulated by the time and temperature of the creaming heat treatment.

Its composition is as follows: 45-70% SiO_2_, 8-20% MgO, 8-15% MgF_2_, 5-35% R_2_O + RO, where R_2_O has a range between 5-25% and is composed of at least one of the following oxides: 0-20% K_2_O, 0-23% Rb_2_O and 0-25% Cs_2_O to improve translucency and RO, which has a range between 0-20%, and is composed of at least one of the following oxides: SrO, BaO and CdO. Additional components may account for up to 10% of Sb_2_O_5_ and/or up to 5% of traditional glassy colorants.[12,13]

There are two important aspects to the formation of the crystalline phase: crystal nucleation and crystal growth. The thermal treatment known as ceraming[14] is composed of two processes: glass is heated up to a temperature where nuclei form (750°–850°C), and this temperature is kept for a period of time ranging from 1 to 6 h so that crystalline nuclei form in the glass (process known as nucleation). Then, the temperature is increased to the crystallization point (1000°–1150°C) and this temperature is maintained for a period ranging from 1 to 6 h until the desired level of glazing is obtained (process known as crystallization).[12,15]

### Composition category 2 – glass-based systems with fillers

#### Leucite-reinforced feldspar glass ceramics

Glass-based systems are made from materials that contain mainly silicon dioxide (also known as silica or quartz), which contains various amounts of alumina.

Aluminosilicates found in nature, which contain various amounts of potassium and sodium, are known as feldspars. Feldspars are modified in various ways to create the glass used in dentistry. Synthetic forms of aluminosilicate glasses are also manufactured for dental ceramics.[16,17]

Pressed glass ceramics are materials containing high amounts of leucite crystals (35% by volume).[14] The basic component of this ceramic is feldspathic porcelain, consisting of 63% SiO_2_, 19% Al_2_O_3_, 11% K_2_O, 4% Na_2_O and traces of other oxides. Leucite crystals are added to the aluminum oxide.[18,19]

This material is manufactured using a process known as heat pressing, which is performed in an investment mold. This mold is filled with the plasticized ceramic thus avoiding the sintering process and the subsequent pore formation.[20] This ceramic undergoes dispersion strengthening through the guided crystallization of leucite.

Dispersion strengthening is a process by which the dispersed phase of a different material (such as alumina, leucite, zirconia, etc.) is used to stop crack propagation as these crystalline phases are more difficult to penetrate by cracks.[14,21]

Leucite crystals are incorporated during ceraming and hence performing this process again is unnecessary when inducing crystal growth.[19]

The construction of ceramic restorations using leucite-reinforced feldspars can be done either by sintering, using a modified version of the sintering process described earlier to construct the porcelain jacket crown, or by a process known as hot pressing.

## LITHIUM DISILICATE AND APATITE GLASS CERAMICS

In order to be able to extend the use of resin-bonded ceramic restorations and possibly use them for bridge construction, a glass ceramic based on a SiO_2_–Li_2_O system has been developed (Empress II, Ivoclar-Vivadent). To increase the strength, thermal expansion and contraction behavior of ceramics, manufacturers have added crystalline filler particles.[22] Other types of filler additions include particles of high-melting glasses that are stable at the firing temperature of the ceramic.[23] Kelly[22] refers to a ceramic as a “glass-ceramic” when the filler particles are added mechanically during manufacturing precipitate within the starting glass by special nucleation and growth-heating treatments. The crystalline phase that forms is a lithium disilicate (Li_2_Si_2_O_5_) and makes up about 70% of the volume of the glass ceramic. Lithium disilicate has an unusual microstructure, in that it consists of many small interlocking plate-like crystals that are randomly oriented. This is ideal from the point of view of strength because the needle-like crystals cause cracks to deflect, branch or blunt; thus, the propagation of cracks through this material is arrested by the lithium disilicate crystals, providing a substantial increase in the flexural strength.

A second crystalline phase, consisting of a lithium orthophosphate (Li_3_PO_4_) of a much lower volume is also present. The mechanical properties of this glass ceramic are far superior to that of the leucite glass ceramic, with a flexural strength in the region of 350–450 MPa and fracture toughness approximately three-times that of the leucite glass ceramic. The glass ceramic is claimed to be highly translucent due to the optical compatibility between the glassy matrix and the crystalline phase, which minimizes internal scattering of the light as it passes through the material [Figures 1–5].

**Figures:**
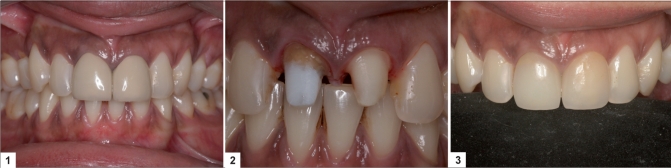
**(1)** 11, 21 metal ceramic crowns with lack of translucency, **(2)** Tooth preparation for glass–ceramic crowns, **(3)** Final IPS Empress 2 crowns showing better translucency

**Figures:**
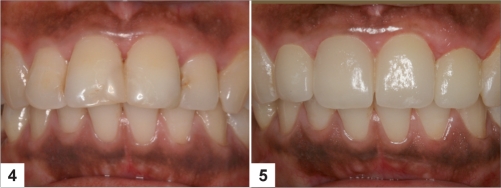
**(4)** Upper anteriors with crowding and multiple composite restorations, **(5)** Upper anteriors restored with glass–ceramic veneers

The processing route is the same as the hot-pressing route described above, except that the processing temperature, at 920°C, is lower than for the leucite glass ceramic. The grain sizes of lithium metasilicate crystals range from 0.2 *µ*m to 1 *µ*m, rendering a flexural strength of 130 MPa to this material. This is comparable to the other mill-ready leucite-reinforced CAD/CAM (ProCAD, Ivoclar Vivadent) blocks and the feldspathic CAD/CAM blocks (Vitabloc Mark II).[24]

During the crystallization cycle, there is a controlled growth of the grain size (0.5–5 *µ*m). This transformation leads to a glass ceramic that is made up of prismatic lithium disilicate dispersed in a glassy matrix.[25] This alteration increases the flexural strength of the restoration to 360 MPa,[26] an increase of 170%. A random orientation of small interlocking plate-like crystals makes up the lithium-disilicate restoration. The orientation and size of the crystals can account for crack deflection and blunting, which, in turn, accounts for the increase in fracture toughness over the leucite-reinforced ceramics.[27]

There are two basic fabrication methods. The first method is to mill the restoration to full anatomical contour. Before crystallization, the incisal edge is preserved by creating a silicone index. The incisal edge is cut back, creating mamelons, and is layered with the appropriate incisal porcelains back to the original contour using the silicone index as the guide. The restoration is then crystallized in the furnace using the standard firing program. A variation of this technique is crystallizing before the layering steps. This method allows the operator to see the colour of the restoration before application of the layering ceramics. This does require a wash coat firing of the layering ceramic before the build-up ceramic is applied.

The second method is to mill the crown to full contour, then stain, glaze and crystallize. This method also has a variation that includes applying the stain and glaze after the crystallization step. This allows the operator to see the final color of the crown while applying the stains. It may be easier to apply the stains, but it involves a second 12-min firing cycle.

### Composition category 3 – crystalline-based systems with glass fillers

#### Glass-infiltrated high-strength ceramic core systems

The addition of alumina to the feldspathic glass during the pre-fritting process limits the amount of alumina that can be incorporated to about 40–50 vol.%. An alternative approach has been adopted in a system called In-Ceram (Vita). This core material has an alumina content of 85%. A ceramic core is formed onto a refractory die from a fine slurry of alumina powder by a process known as slip casting. After the die has dried, it is sintered for 10 h at 1120°C. The melting temperature of alumina is too high to produce full densification of the powder by liquid phase sintering, and solid phase sintering alone occurs. Consequently, the coping thus created is only just held together at the contact points between the alumina particles, and a porous structure is the result. The strength of this porous core is only about 6–10MPa. The porous structure is then infiltrated with a lanthanum glass, which has a low viscosity when fired at 1100°C for 4–6 h, which increases the strength. The molten glass is able to penetrate into the pores, producing a dense ceramic. The esthetics and functional form are then achieved by the use of conventional feldspathic dental ceramics.[28,29]

## IN-CERAM SPINELL, ALUMINA, ZIRCONIA

Infiltrated ceramics are made through a process called slip-casting, which involves the condensation of an aqueous porcelain slip on a refractory die. This fired porous core is later glass infiltrated, a process by which molten glass is drawn into the pores by capillary action at high temperatures. Materials processed in this way exhibit less porosity, fewer defects from processing, greater strength and higher toughness than conventional feldspathic porcelains.[29]

This glass-infiltrated core is later veneered with a feldspathic ceramic for final esthetics. These have excellent translucency and esthetic qualities, but have poor physical properties and require the high-strength core that the already-mentioned infiltrated ceramics can provide. The Vita In-Ceram slip-casting system makes use of three different materials to gain a good compromise between strength and esthics.

### In-Ceram spinell

Spinell (MgAl_2_O_4_) is a natural mineral that is normally found together with limestone and dolomite. It is of dental significance because of its extremely high melting point (2135°C) combined with its high strength. Spinell is also chemically inert and has low electrical and thermal conductivity but, most importantly, it has unique optical properties. It has moderate strength of about 350 MPa and good translucency.

It is more than twice as translucent as In-Ceram alumina due to the refractive index of its crystalline phase being close to that of glass. Glass infiltrating in a vacuum environment results in less porosity, ensuring this high level of translucency. Often, however, this level of translucency can be excessive and can lead to an overly glassy, low-value appearance.

### In-Ceram alumina

Aluminum oxide (Al_2_O_3_) is most widely known under the term corundum. As a result of the homogeneous framework structure made of ultrafine Al_2_O_3_ particles, whose cavities are filled with a special glass, the degree of tensile bending strength is significantly higher than that of all other ceramic systems.[1] With a weight percentage of 10–20%, aluminum oxide is a component of feldspar, which is the starting material for metal–ceramic veneering materials. The ceramic materials for substructures of jacket crowns have been enriched by up to 60% by weight with aluminum oxide crystals with a grain size of 10–30 um to increase stability. Because of the large difference in the refraction index (feldspar *n* = 1.53; corundum *n* = 1.76), intense refraction of light occurs at the aluminum oxide crystals in the feldspar, which results in the opaque effect of such Al_2_O_3_-enriched ceramic materials. Therefore, they are only suitable for fabrication of crown frames with subsequent veneering. In-Ceram alumina has a strength of around 500 MPa and poor translucency.

Synthetically produced corundum with a grain size of 2–5 um is used for In-Ceram alumina. In the solid phase, it is sintered at 1100°C, well below the melting point of 2040°C, and it is then infiltrated with dentine-coloured glass at 1120°C.

### In-Ceram zirconia

The zirconia system uses a mixture of zirconium oxide and aluminum oxide as a framework to achieve a marked increase in the flexural strength in the core framework. Aluminum oxide makes up about two-thirds of the crystalline structure as seen in the scanning electron micrograph to the right. The remaining crystalline structure consists of tetragonal zirconium oxide (round white particles). The proportion of glass phase amounts to approximately 20–25% of the total structure. This leads to the high strength as already seen in In-Ceram alumina. The increase however over alumina is due to the zirconium oxide particles that protect the structure against crack propagation. It has a very high strength of around 700 MPa and very poor translucency.

## COMPOSITION CATEGORY 4 – POLYCRYSTALLINE SOLIDS

### Polycrystalline ceramics

#### Transformation-toughened zirconium oxide

Zirconia occurs as a natural mineral called baddeleyite. This mineral contains 80–90% zirconium oxide. The major impurities are usually TiO_2_, SiO_2_ and Fe_2_O_3_. This oxide exists in three different crystal structures: monoclinic at room temperature, tetragonal at ~1200°C and cubic at 2370°C. Zirconium oxide is transformed from one crystalline state to another during firing. At the firing temperature, zirconia is tetragonal and at room temperature, it is monoclinic, with a unit cell of monoclinic occupying about 4.4% more volume than when tetragonal. Unchecked, this transformation was unfortunate because it led to crumbling of the material on cooling.

In the late 1980s, ceramic engineers learned to stabilize the tetragonal form at room temperature by adding small amounts (3–8 mass%) of calcium and later yttrium or cerium. Although stabilized at room temperature, the tetragonal form is “metastable,” meaning that trapped energy exists within the material to drive it back to the monoclinic state. The highly localized stress ahead of a propagating crack is sufficient to trigger grains of ceramic to transform in the vicinity of that crack tip. In this case, the 4.4% volume increase becomes beneficial, essentially squeezing the crack closed (i.e., transformation decreases the local stress intensity).[30–32]

Two key developments allowed fully polycrystalline ceramics to become practical for fixed prostheses:

1. The availability of highly controlled starting powders and
2. the application of computers to ceramics processing.

Unlike glassy ceramics, polycrystalline ceramics cannot be pressed as a fully dense material into slightly oversized molds (molds that have expanded just enough to compensate for cooling shrinkage, as is done in the casting of metals). Polycrystalline ceramics are formed from powders that can be packed only to 70% of their theoretical density. Hence, polycrystalline ceramics shrink around 30% by volume (10% linear) when made fully dense during firing. For the final prostheses to fit well, the amount of shrinkage needs to be accurately predicted and compensated for. Well-characterized starting powders that can be uniformly packed are a prerequisite for achieving predictable and reproducible shrinkage.

Two approaches are offered commercially for fabrication of prostheses from polycrystalline ceramics, both of which create oversized greenware (unfired part) using 3-D data sets and the specific shrinkage characteristics of well-behaved starting powders.

1. In the first approach, an oversized die is manufactured based on 20,000 measurements taken during the mechanical scanning of a laboratory die. Aluminum oxide or zirconium oxide is pressed onto the oversized die and predictably shrunk during firing to become well-fitting, single-crown substructures (Procera, Nobel Biocare).[33]
2. In the second approach, blocks of partially fired (10% complete) zirconium oxide are machined into oversized greenware for firing as single- and multiple-unit prostheses substructures (Cercon, Dentsply Prosthetics; Lava, 3M-ESPE; YZ, Vita). In these systems, individual blocks are bar coded with the actual density of each block (for the fine-tuning of shrinkage calculations), and the milling machines can keep track of the number of blocks milled and automatically change milling tools to assure accuracy of fit.[34]

## CAD/CAM TECHNOLOGIES AND MATERIALS

### Concept overview

Utilizing in-office CAD/CAM technology, clinicians can design, fabricate and place all-ceramic inlays, onlays, crowns and veneers in a single patient visit. The ceramic restorations produced by this method have demonstrated excellent fit, strength and longevity.

Two basic techniques can be used for CAD/CAM restorations.

1. Chairside single-visit technique.
2. Integrated chairside–laboratory CAD/CAM procedure.

## CHAIRSIDE CAD/CAM TECHNIQUE

### The CEREC system

The CEREC system provides an in-office alternative for porcelain restorations. The processing begins with a smooth, rounded, well-tapered restoration. This preparation is sprayed and bonded to titanium dioxide contrast powder in the patient’s mouth. An infrared camera records the powder and creates a 3-D optical impression on the computer. This image can be manipulated by the dentist to create ideal anatomy and contacts before processing. The shade of porcelain is selected by the dentist, and this shade selection is placed into the computer. The computer then tells the dentist what block of porcelain or composite is to be used. This block is then milled in-office according to the computer design. The restoration comes out of the milling machine with a ceramic sprue that needs to be removed. The restoration is then tried in the patient’s mouth.

Proximal contacts may need to be adjusted and flash may need to be removed. If the restoration is adequate and esthetic, it can be cemented in using composite.

### Integrated chairside–laboratory CAD/CAM technique

An integrated chairside–laboratory technique requires two visits. The clinician either can scan the preparation directly and then send the scan to the laboratory or can take a traditional impression, after which a stone model is poured and the laboratory scans the stone model. In the first case, the patient still does not require an impression, removing a source of discomfort for the patient and a potential source of inaccuracy for the clinician.

## RESTORATION DESIGN AND FABRICATION

Designing the virtual restoration is similar to that traditionally performed at the laboratory: the die margins are trimmed and the restoration is designed. Rather than physically building up the restoration using layers of porcelain, however, the clinician is presented with a fully contoured 3-D model of the restoration to refine.

By accessing a comprehensive database of natural tooth structures, the clinician is able to assess the design proposed by the computer and to verify its fit in relation to the preparation, the gingival margins and the neighbouring teeth, as well as the occlusion.

Any refinements deemed necessary by the dentist are accomplished with the computer software’s design tools. Using the CAD software, the practitioner can form the desired interproximal contacts and verify occlusal relationships prior to milling. The image of the restoration on the computer screen would be reproduced in the milling process.

Using a CAD/CAM restorative technique, a number of steps can be simplified or eliminated. Traditional impressions can be replaced by using a handheld scanning device that digitally records the form and margins of the preparation. Care must be taken to ensure that the whole preparation is scanned, to avoid introducing errors. As with a traditional impression, soft-tissue retraction and hemostasis are prerequisites for an accurate result.

## MATERIAL OPTIONS FOR THE CAD/CAM TECHNIQUE

Advances in dental ceramic materials and processing techniques, specifically CAD/CAM and milling technology, have facilitated the development and application of superior dental ceramics. CAD/CAM allows the use of materials that cannot be used by conventional dental processing techniques. Tightly controlled industrial ceramic processing can produce increased microstructural uniformity, higher density, lower porosity and decreased residual stresses. Such improvements have the potential to improve clinical predictability.

CAD/CAM has become somewhat synonymous with zirconia, but systems are available that can machine any type of ceramics, i.e. glass ceramics, interpenetrating (infiltration ceramics) materials and solid-sintered monophase ceramics such as zirconia.

The material used depends on functional and esthetic demands and on whether a chairside or laboratory CAD/CAM restoration is fabricated. For chairside CAD/CAM restorations, an esthetic, strong material requiring minimal post-milling esthetic adjustment to minimize the chairside time is needed. Leucite-reinforced glass ceramics (IPS Empress CAD, Ivoclar Vivadent;) and lithium disilicate glass ceramics (IPS e.max, Ivoclar Vivadent) can be used for chairside and laboratory CAD/CAM single restorations. Leucite-reinforced material is designed to match the dentition for strength and surface smoothness and to offer esthetic results by scattering light in a manner similar to enamel.

For chairside cases where strength is a consideration, lithium disilicate CAD restorations offer a strength of 400 MPa as compared with the leucite-reinforced ceramic, with an MPa ranging from 120 to 160, and still provide good esthetics. Lithium disilicate is used as a monolithic (single layer) material, providing strength.

## CAD/CAM AND SOLID-SINTERED MONOPHASE CERAMICS

Solid-sintered ceramics have the highest potential for strength and toughness but, because of high firing temperatures and sintering shrinkage techniques, they were not available to use as high-strength frameworks for crowns and fixed partial dentures until recently. Solid-sintered monophase ceramics are materials that are formed by directly sintering crystals together without any intervening matrix to form a dense, air-free, glass-free, polycrystalline structure. There are several different processing techniques that allow the fabrication of either solid-sintered aluminous oxide or zirconia oxide frameworks.

There are three basic techniques for fabricating solid-sintered monophase ceramic frameworks for porcelain application.

1. One system, DCS Preciscan (marketed in the United States by Dentsply) machines the final desired framework shape from a solid-sintered block of material. This system is expensive and has not proven cost-effective as a result of the excessive machining time and manual labour necessary to adjust and fit the coping.
2. Secondly, the Procera^®^ system (Nobel Biocare) employs an oversized die where slurry of either aluminous oxide or zirconia oxide is applied to the die, subsequently fired, fully sintered and shrunk to fit the scanned die.
3. The third method machines an oversized coping from a partially sintered block of zirconia oxide material, which is then fired to the full sintering temperature and then shrunk to fit the die. Most of the systems on the market today use some variation of this type of technology. Examples of these systems are Lava™ (3M™ESPE™) and Cercon (Dentsply). These systems scan the prepared die and then the software creates virtual dies and frameworks. An oversized framework is created through a CAM process, which is then fully sintered in a special oven. The Lava™ systems also allow for internal shading of the core material [Figures 6–8].

**Figures:**
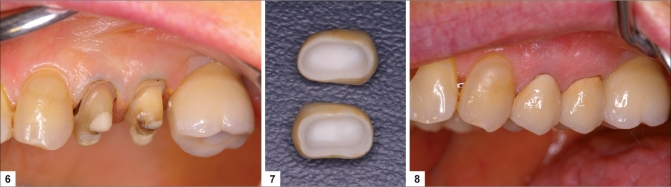
**(6)** Tooth preparation for monophase zirconia fixed partial denture, **(7)** Zirconia framework (Lava 3MESPE), **(8)** Completed restoration immediately post-op

## STRENGTH AND FRACTURE TOUGHNESS

There are two interrelated properties that often are quoted regarding ceramics intended for structural purposes:

1. Strength and
2. Fracture toughness

### Strength

Mechanical failure of ceramic materials is almost completely controlled by brittle fracture. Usually, this brittle behavior combined with surface flaws resulted in relatively low ceramic strengths. Increased crystalline-filler content within the glass matrix, with a more even distribution of particles and finer particle size, has yielded significant improvements in the flexural strength of ceramic materials.[35,36] However, strength improvements are still limited by the inherent weakness of the glass matrix. All ceramics fail because of crack propagation at a critical strain of 0.1%.[37] Applied stresses can cause a crack to grow throughout the matrix, causing the ultimate failure of that restoration.

### Fracture toughness

A more important physical property is fracture toughness, which has been reported to be between 8 MPa m1/2 and 10 MPa m1/2 for zirconia. This is significantly higher than any previously reported ceramic, and roughly twice the amount reported for the alumina materials. Fracture toughness is a measure of a material’s ability to resist crack growth (i.e., a measure of the amount of energy necessary to cause crack growth). Clinically, restorations are not loaded to failure as is done in a flexural strength test; instead, millions of subcritical loads (chewing) are applied. Materials ultimately fail because of this cyclic fatigue by crack propagation. Thus, materials with higher fracture toughness are more ideal clinically as it takes more energy to cause crack growth. Other factors such as stress corrosion (chemically assisted crack growth) and residual flaws in the material greatly affect the final strength of a finished material.[38,39]

Mechanisms that can lead to toughened or strengthened ceramics can be categorized into the following three types:

#### Crack tip interactions

These occur when obstacles in the microstructure act to impede the crack motion. These are generally second-phase particles and act to deflect the crack into a different plane so that it is no longer subject to the normal tensile stress that originally caused its propagation.

#### Crack tip shielding

These are a result of events that are triggered by high stresses in the crack tip region that act to reduce these high stresses. Transformation toughening and microcrack toughening are two mechanisms that have been identified as leading to crack tip shielding.

#### Crack bridging

This occurs when the second-phase particles act as a ligament to make it more difficult for the cracks to open. Crack bridging is best understood for bonded fiber composites. This mechanism has been shown to be important in large-grain Al_2_O_3_ and possible whisker-reinforced ceramic materials.

## CONCLUSION

The new generation of ceramic materials present interesting options, both in terms of material selection and in terms of fabrication techniques. A closer understanding of the dynamics of the materials with respect to design of the restoration and the intended use is required to enable these restorations to perform productively.
